# Supplementary material for: Dyskerin Downregulation Can Induce ER Stress and Promote Autophagy via AKT-mTOR Signaling Deregulation
Source: Biomedicines. 2022 May 8;10(5):1092. doi: 10.3390/biomedicines10051092 (PMC9138296; doi:10.3390/biomedicines10051092)
Supplement: Supplementary file 1 [file biomedicines-10-01092-s001.zip › Figure S1.pdf]

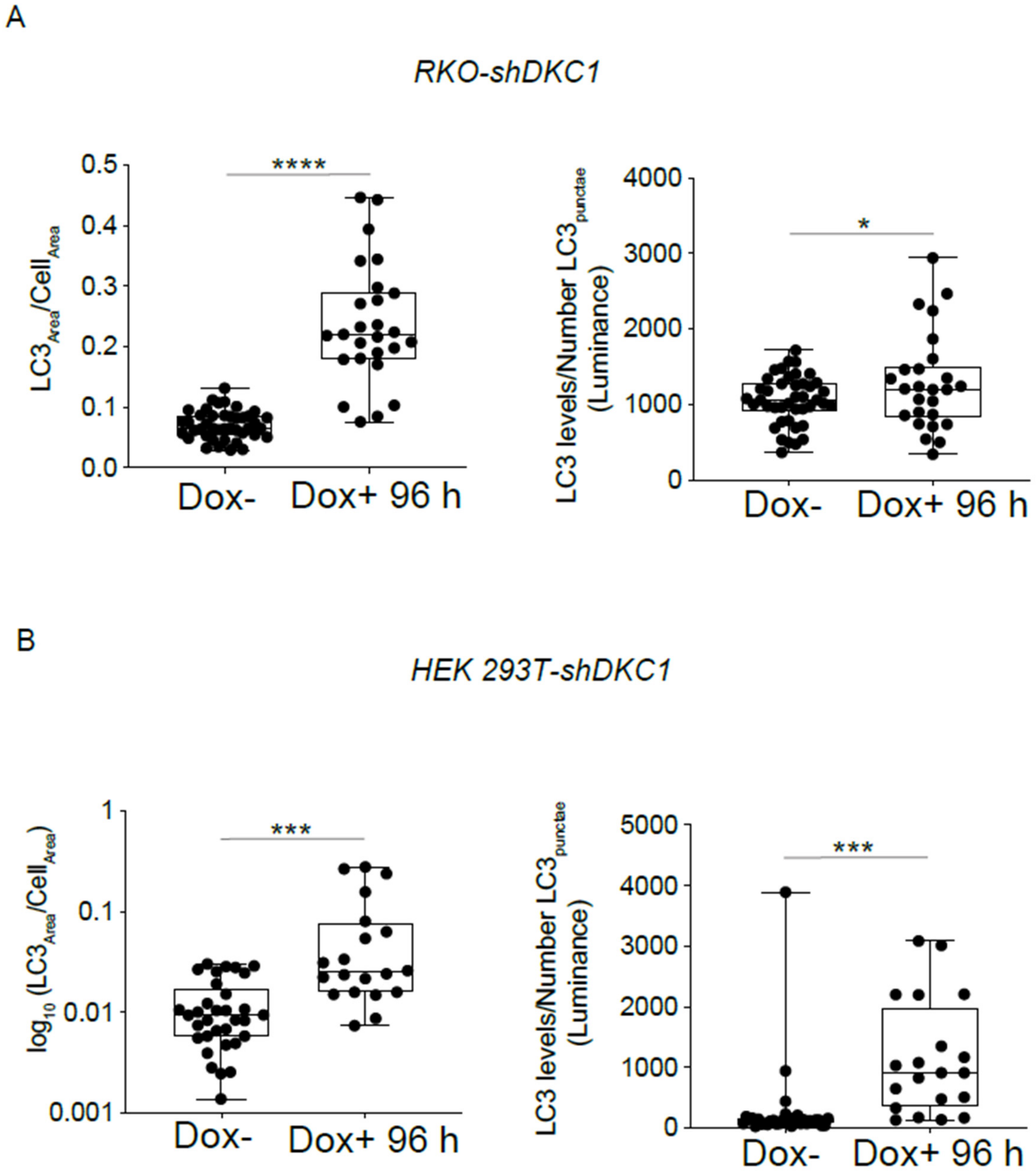

Figure S1. Dyskerin depletion increases LC3 cytoplasmic levels. (**A and B**) Quantification of LC3 immunoreactivity from confocal micrographs of control (Dox-) and silenced (Dox+) RKO-shDKC1 (**A**) and HEK 293T-shDKC1 (**B**) cells, immunolabelled with anti-LC3 and anti-dyskerin antibodies. Nuclei were counterstained with DAPI. Increased LC3 immunoreactivity upon dyskerin depletion is documented by the increased LC3Area/CellArea ratio and by the increased amount of LC3 fluorescence per puncta (calculated as [RawIntDen/LC3Area]/Number LC3puncta). Values are expressed as mean ± SEM (N = 42 RKO-shDKC1 Dox-; N = 26 RKO-shDKC1 Dox+ 96 h; N = 33 HEK 293T-shDKC1 Dox-; N = 20 293T HEK 293T-shDKC1 Dox+ 96 h). An unpaired one-tailed t-test was used in pairwise comparisons respect to the Dox- condition for statistical analysis (**** $p < 0.0001$; *** $p < 0.001$; ** $p < 0.01$; * $p < 0.05$; ns, $p > 0.05$). .
